# Costs analysis of radiotherapy for breast cancer in Indonesia: a comparison between reimbursement tariffs and actual costs

**DOI:** 10.1186/s12913-025-12849-9

**Published:** 2025-05-28

**Authors:** Fithria Dyah Ayu Suryanegara, Deni Iskandar, Ericko Ekaputra, Eko Kuntjoro, Didik Setiawan, Maarten Jacobus Postma, Lisa Aniek de Jong

**Affiliations:** 1 Department of Health Sciences, University of Groningen, University Medical Center Groningen, Groningen, The Netherlands; 2 Department of Pharmacy, Universitas Islam Indonesia, Yogyakarta, Indonesia; 3 Faculty of Pharmacy, Bhakti Kencana University, Bandung, Indonesia; 4 Department of Radiology, Faculty of Medicine, Public Health and Nursing, Universitas Gadjah Mada, Yogyakarta, Indonesia; 5 Rumah Sakit Umum Pusat Dr. Sardjito, Yogyakarta, Indonesia; 6 Ken Saras Hospital, Semarang, Indonesia; 7 Faculty of Pharmacy, Universitas Muhammadiyah Purwokerto, Purwokerto, Indonesia; 8 Center of Excellence for Pharmaceutical Care Innovation, Universitas Padjadjaran, Bandung, Indonesia; 9 Department of Economics, Econometrics & Finance, Faculty of Economics & Business, University of Groningen, Groningen, The Netherlands; 10 Division of Pharmacology and Therapy, Department of Anatomy, Histology, and Pharmacology, Faculty of Medicine, Universitas Airlangga, Surabaya, Indonesia

**Keywords:** Breast cancer, Radiotherapy, Reimbursement, Cost analysis, Hospital administration

## Abstract

**Background:**

Breast cancer is the most common cancer in Indonesia, and radiotherapy plays an essential role in its treatment. However, since 2016, the INA-CBGs (Indonesian Case-Based Groups) tariffs for radiotherapy have remained unchanged. This study aimed to assess the disparity between tariffs and actual costs of outpatient radiotherapy in breast cancer, using real-world data from two Indonesian hospitals.

**Methods:**

We conducted a retrospective cohort study in a national public referral hospital and a private hospital. Breast cancer claims data were collected from 2017 to 2022 from the Department of Accounting/Finance with INA-CBGs tariff code of C-3-10-0 (radiotherapy procedures for outpatients). We estimated total actual costs, actual costs per patient and visit, and the cost-tariffs ratio. Differences between the actual costs and tariffs were analyzed using Mann-Whitney test.

**Results:**

A total of 3,890 breast cancer patients were included in the study, of which 74.4% were from the national public referral hospital. In the national public referral hospital and private hospital, the total actual costs of outpatient radiotherapy in breast cancer were USD 19,028,791.17 and USD 5,279,980.74, with median costs per patient of USD 6,560.00 [3,679.81;7,518.46] and USD 5,110.00 [839.15;7,552.34], and median costs per visit of USD 272.00 [253.16;274.47] and USD 272.00 [211.31;305.50], respectively. Over the study period, the cost-tariffs ratio was 86.85% and 59.07% in the national public referral hospital and private hospital, respectively. The differences between the tariffs and total actual costs were statistically significant in both hospitals and increased throughout the years.

**Conclusions:**

For both hospitals, the INA-CBGs tariffs for outpatient radiotherapy services for breast cancer were insufficient to fully cover the actual costs during the review period. Furthermore, the difference between the tariffs and the actual costs increased over the years, emphasizing the need for revision of the C-3-10-0 tariffs. It is crucial to ensure coverage of all actual costs to ensure the sustainability, accessibility, and availability of radiotherapy treatment for breast cancer patients in Indonesia.

**Supplementary Information:**

The online version contains supplementary material available at 10.1186/s12913-025-12849-9.

## Introduction

Cancer is a catastrophic disease with a high economic burden, accounting for 18% of Indonesia’s National Health Insurance Program (NHIP) budget. According to a report by BPJS Kesehatan (*Badan Penyelenggara Jaminan Sosial*/Social Security Administrator for Health), which manages the NHIP, cancer is the second largest economic burden after cardiovascular disease [1]. This economic burden is expected to increase alongside the expected increase in cancer epidemiology [2].

Breast cancer is the most common cancer in Indonesia, with an estimated number of new cases of around 66,271 in 2022, and is expected to increase by almost 40% in 2045 [3]. Furthermore, breast cancer mortality rates in Indonesia exceed the global rate of 12.7 per 100,000, with an estimated rate of 14.4 per 100,000 [4]. With the high mortality rate of breast cancer in Indonesia relative to the global rate, improved breast cancer treatment management is necessary.

Radiotherapy plays an important role in preventing the recurrence and mortality of breast cancer patients [5, 6]. A meta-analysis has shown that radiotherapy reduces the 10-year risk of recurrence by 15.7% and the 15-year risk of breast cancer death by 3.8% [7]. Nevertheless, radiotherapy continues to be marginalized in terms of healthcare financing. Only 5% of the annual cancer care budget and 0.5% of the total healthcare budget in European countries are spent on radiotherapy [8].

In Indonesia, under the NHIP, radiotherapy is reimbursed prospectively per patient per visit by BPJS Kesehatan to the healthcare provider. Reimbursement is regulated under the Indonesian Case-Based Groups (INA-CBGs) tariffs, which include all resources used in the diagnosis and treatment of diseases with similar clinical characteristics [9]. However, the tariffs for radiotherapy in breast cancer have remained unchanged since 2016 [10].

Reimbursement is a key factor in ensuring sustainability, accessibility, and availability of resources in a health system that provides high-quality care [8]. To ensure tariffs cover current standards of care, reimbursement terms, and tariffs should be reviewed from time to time. A previous study has shown the need to reevaluate INA-CBGs tariffs for inpatient care for high-incidence cancers, including breast cancer [11], which could also be the case for INA-CBGs tariffs for outpatient radiotherapy [10, 12]. To assess whether the INA-CBGs tariffs adequately cover the actual costs of radiotherapy for breast cancer outpatients, we conducted a retrospective cohort study using real-world data from two Indonesian hospitals. We estimated the total actual costs, actual costs per patient and visit, and the cost-tariffs ratio of radiotherapy in both hospitals.

## Methods

### Study design

We conducted a retrospective cohort study to collect data on the actual costs of radiotherapy in two hospitals in Indonesia. One hospital concerns a national public referral hospital of type A located in Yogyakarta Special Province (hereafter referred to as Public Hospital A), and the second hospital concerns a private hospital of type C located in Central Java Province (hereafter referred to as Private Hospital C). The classification of hospitals in types A and C refers to the Ministry of Health of the Republic Indonesia regulation, in which the hospital type is based on the number of beds for inpatients (A ≥ 250 beds, B 200–249 beds, C 100–199 beds, and D 50–99 beds) [13]. We applied convenience sampling to select the hospitals included in the study. Public Hospital A and Private Hospital C were selected based on their roles in providing radiotherapy services for breast cancer patients in Regional Division VI BPJS Kesehatan. Public Hospital A, a national referral hospital in Indonesia, is located in Yogyakarta Special Province, which has the highest cancer incidence in Indonesia. Private Hospital C is located in Central Java Province and provides radiotherapy services in partnership with BPJS Kesehatan.

We analyzed total actual costs, actual costs per patient and visit, and the cost-tariffs ratio based on claims data from 2017 to 2022 for breast cancer outpatients who underwent at least one course of radiotherapy. These data were obtained from claims data for the INA-CBGs code of C-3-10-0 (radiotherapy procedures for outpatients), which were provided by the Department of Accounting of Public Hospital A and the Department of Finance of Private Hospital C. As these data were submitted to BPJS Kesehatan for reimbursement purposes, they should be valid. The claims data included information on patient characteristics, primary and secondary diagnoses using International Classification of Disease Tenth Revision (ICD-10) codes, clinical procedures using ICD-9 codes, radiotherapy visits, cost components (e.g. radiotherapy procedures, consultation, medicines, medical consumable, etc.), total actual costs, and total INA-CBGs tariffs received. All the personal data of the patients were deidentified before the analysis to ensure confidentiality. The study was reviewed and approved by the Medical Health Research and Ethics Committee of the Faculty of Medicine, Public Health, and Nursing Universitas Gadjah Mada (approval number KE/FK/0449/EC/2018 and KE/FK/1413/EC/31 December 2021). The reporting of this study followed the Strengthening the Reporting of Observational Studies in Epidemiology (STROBE) checklist [14].

### Patients

Patients had to be members of NHIP with primary and secondary diagnoses of either breast cancer (ICD-10 code C50.9; malignant neoplasm of breast unspecified) or radiotherapy (ICD-10 code Z51.0; radiotherapy sessions) and vice versa, linked to claims data for INA-CBGs code C-3-10-0 (outpatient radiotherapy procedures). We excluded patients with any missing cost data from the analysis.

### INA-CBGs tariffs

The radiotherapy reimbursement system in Indonesia is regulated by the Ministry of Health of the Republic of Indonesia Decree No. 3-year of 2023 about health services tariffs in the national health insurance program. The INA-CBGs tariffs represent the maximum amount of claims payment by the BPJS Kesehatan (Social Security Administrator for Health) to the referral health services providers (e.g., hospitals type A, B, C, and D) on the service packages based on the grouping of disease diagnoses and procedures [12]. The INA-CBGs tariffs are differentiated based on hospital type, the ownership of hospitals (public versus private), region, type of disease, and severity of the disease [12]. It concerns a prospective payment system, where tariffs are determined before the intervention is delivered to the patients. INA-CBGs tariffs only cover direct medical costs such as medicine costs, laboratory costs, physician costs, etc., and cannot cover direct non-medical costs and indirect costs such as transportation and accommodation.

The latest version of the INA-CBGs tariffs [12] has replaced Decree Number 52 in 2016 [10]. However, the tariffs for outpatient radiotherapy with code C-3-10-0 have not changed in this latest update and remain as much as Indonesian Rupiah (IDR) 1,144,000 (USD 240.53) per session/visit in Public Hospital A and IDR 769,000 (USD 161.68) in Private Hospital C. The tariffs were expressed in the respective price year.

### Actual costs

We defined actual costs as the costs for all hospital resources used for the treatment of breast cancer outpatients receiving radiotherapy for the patients identified in the study under code C-3-10-0. The actual costs consisted of direct medical costs, including radiotherapy procedures, medical consultation with the radiology oncologist, medicines, laboratory examination, accommodation, nurse services, non-surgery procedures, and others (e.g., use of medical consumable and devices).

### Outcomes

Actual costs and INA-CBGs tariffs received were reported as a total, per patient, and visit for each year. The actual costs made by the hospital were compared to the INA-CBGs tariffs received, expressed as absolute difference and as cost-tariffs ratio, which represents the proportion of actual costs covered by the tariffs. Actual costs were also presented for each radiotherapy cost component (radiotherapy procedures, medicine, medical consumable, laboratory, radiology, non-surgery procedure, consultation, nurse, supporting, blood transfusion, accommodation, surgery, and medical device) separately.

Actual costs and INA-CBGs tariffs were expressed in the respective price year. The conversion of IDR to USD was performed using Purchasing Power Parity (PPP), sourced from the World Bank [15].

### Statistical analysis

Descriptive statistics (means and standard deviations (SD), median and interquartile range (IQR) for continuous variables and percentages for discrete variables) were performed to represent the patient’s characteristics. Continuous variables were INA-CBGs tariffs, actual costs, and the number of visits. The discrete variables were the year of visit, gender, and insurance class. Since the actual costs and INA-CBGs tariffs were not normally distributed based on Kolmogorov-Smirnov tests, the differences between actual costs and INA-CBGs tariffs were assessed using a Mann-Whitney test, with a p-value of < 0.05 being considered statistically significant. All analyses were conducted using R version 4.6.0 and SPSS version 28.

### Sensitivity analysis

We conducted several sensitivity analyses to test the robustness of our results. A sensitivity analysis was conducted by forecasting the future value of INA-CBGs tariffs from 2016 to 2017–2022 with adjustment for inflation.

The future value formula:

$$\mathrm{FV = PV\;(in\;n)\;\times\;inflation\;rate\;in\;i/inflation\;rate\;in\;i-1}$$

FV = Future Value

PV = Present Value

i = year i

Additionally, we performed sensitivity analyses to analyze the effect of varying patient demographics on the actual costs and their discrepancy with INA-CBGs tariffs. We classified the patients based on the patient’s age into 2 (two) groups, below 65 years (adults) and ≥ 65 years (elderly). Furthermore, we categorized the patients into 2 (two) classes of insurance: second class and third class. The different classes of insurance were determined by the monthly insurance premium. To assess the differences in tariffs and actual costs in these groups, a statistical analysis was performed using the Mann-Whitney test, with *p* < 0.05 considered significant.

## Results

### Patient characteristics

A total of 3,890 breast cancer patients were included in the study (74.4% from Public Hospital A; 25.6% from Private Hospital C). In Public Hospital A, there was missing cost data for 375 of 69,774 (0.54%) visits. The claims data in Private Hospital C were only available for the years 2019, 2021, and 2022. The 2017–2018 and 2020 data were unavailable because of the absence of registration and machine maintenance in those years. Most patients were female (96.86%), covered by the third insurance class of BPJS Kesehatan (93.70%), with a median age of 51.90 years (SD 10.15). All patients’ characteristics are summarized in Table 1.

**Table 1:** Characteristics of breast cancer outpatients undergoing radiotherapy at Public Hospital A and Private Hospital C

Variable	Public Hospital A	Private Hospital C	Overall
	(*N* = 2,893)	(*N* = 997)	(*N* = 3,890)
Year, n (%)			
2017	318	NA	318
2018	493	NA	493
2019	636	555	1,191
2020	534	NA	534
2021	444	271	715
2022	468	171	639
Age (years), mean ± SD	52.20 ± 10.10	51.10 ± 10.20	51.90 ± 10.15
Gender, n (%)			
Female	2,832 (97.89)	936 (93.88)	3,768 (96.86)
Male	61 (2.11)	61 (6.12)	122 (3.14)
Insurance Class, n (%)			
2^nd^ Class	245 (8.47)	NA	245 (6.30)
3^rd^ Class	2,648 (91.53)	997 (100)	3,645 (93.70)

*N* number of patients, *C50.9* malignant neoplasm of breast unspecified, *Z51.0* radiotherapy sessions, *NA* not available, *SD* standard deviation

### Number of visits and patients

**Fig. 1:**
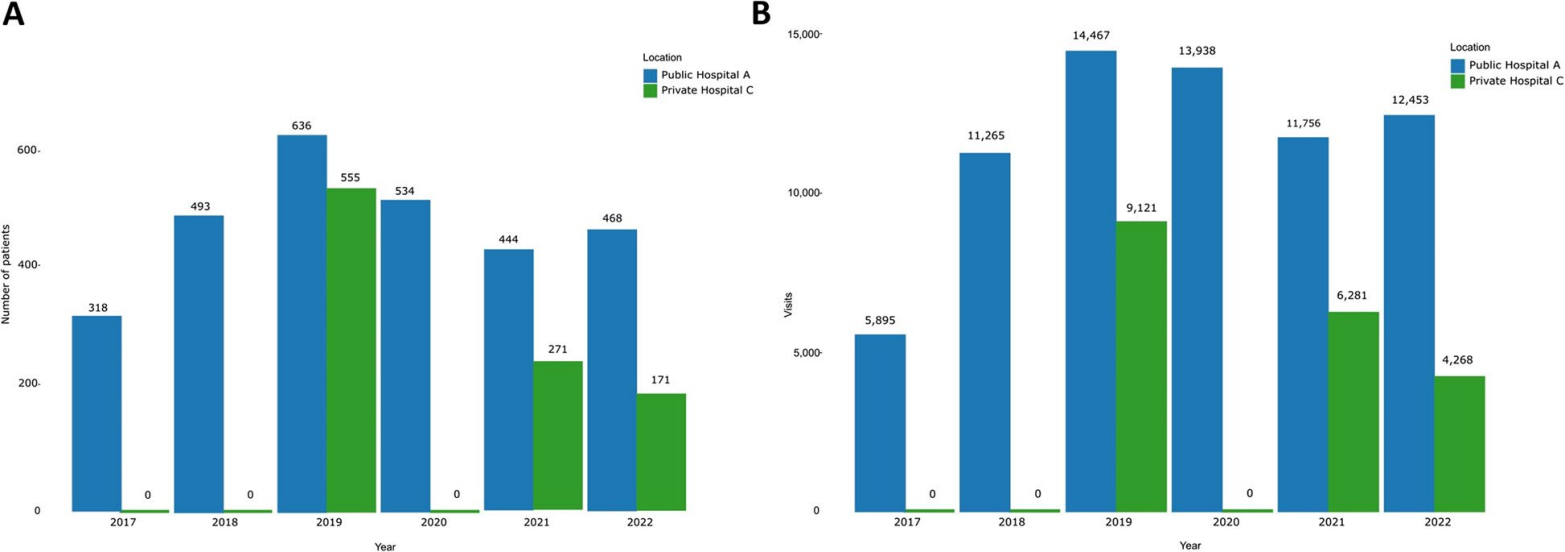
Number of breast cancer patients (**A**) and their visits (**B**) to the radiotherapy department at Public Hospital A and Private Hospital C in the period 2017–2022

During the review period, the total number of visits for both hospitals was 89,414. The number of patients and visits fluctuated in Public Hospital A, with a rising trend from 2017 to 2019, followed by a decline in 2020 and 2021, after which the number again increased in 2022. In Private Hospital C, a decreasing trend in the number of patients and visits was observed over the years (Fig. 1A and B).

### Comparison of actual costs versus INA-CBGs tariffs

**Fig. 2:**
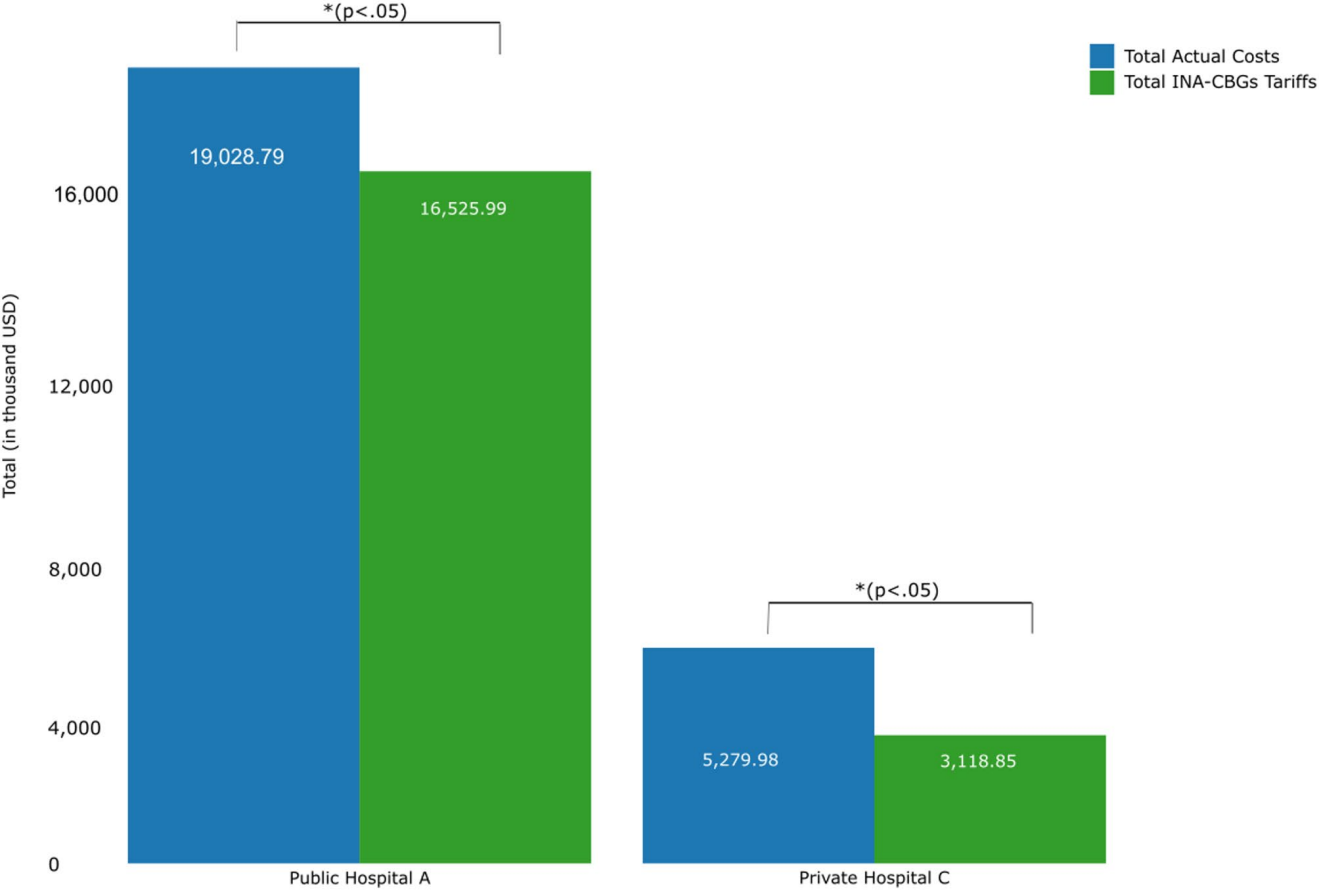
The total actual costs and total INA-CBGs tariffs in both hospitals during the period 2017–2022

**Fig. 3:**
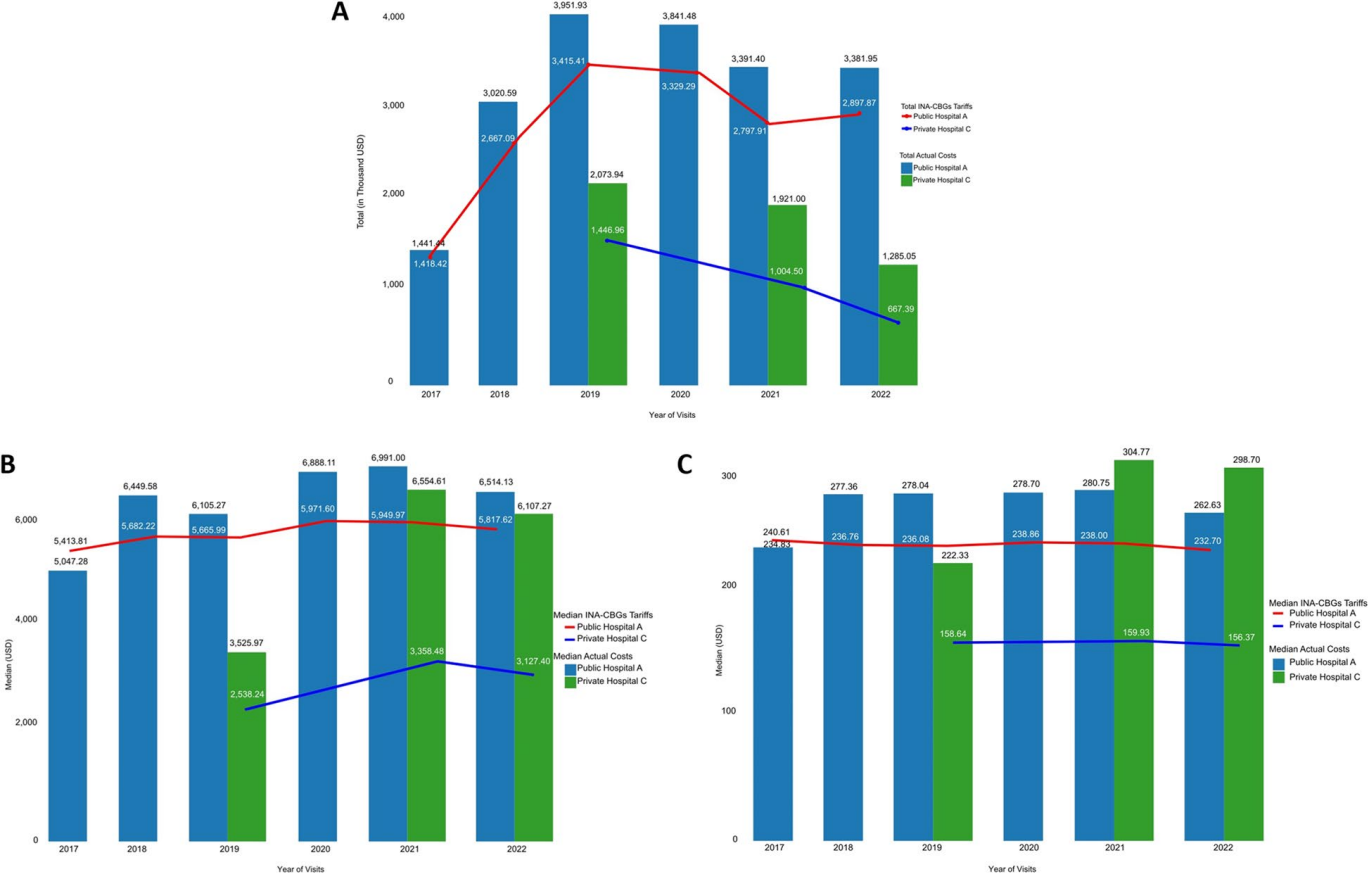
Total actual costs and INA-CBGs tariffs (**A**), median actual costs and INA-CBGs tariffs per patient (**B**), and per visit (**C**) in both hospitals annually

In both hospitals, the total actual costs for breast cancer outpatients with radiotherapy treatment during the review period were significantly higher than the received INA-CBGs tariffs (*p* < 0.05) (Fig. 2). Consistently throughout the review period, INA-CBGs tariffs did not cover total actual costs made by the hospitals for outpatient radiotherapy to treat breast cancer patients, which resulted in total financial losses amounting to (USD 2,502,796.17) in Public Hospital A and (USD 2,161,132.34) in Private Hospital C (Figs. 2 and 3A, and Supplementary Table 1).

On a patient and visit level, similar trends were observed: the INA-CBGs tariffs also never surpassed the actual costs in any of the included years (Fig. 3B and C). The average actual costs per patient and visit in Public Hospital A were higher than in Private Hospital C. Across the entire period, the median actual costs were USD 6,560.00 [3,679.81;7,518.46] per patient in Public Hospital A and USD 5,110.00 [839.15;7,552.34] per patient in Private Hospital C. However, there was not much difference in the median cost per visit between Public Hospital A and Private Hospital C, USD 272.00 [253.16;274.47] and USD 272.00 [211.31;305.50], respectively (Supplementary Table 2).

**Fig. 4:**
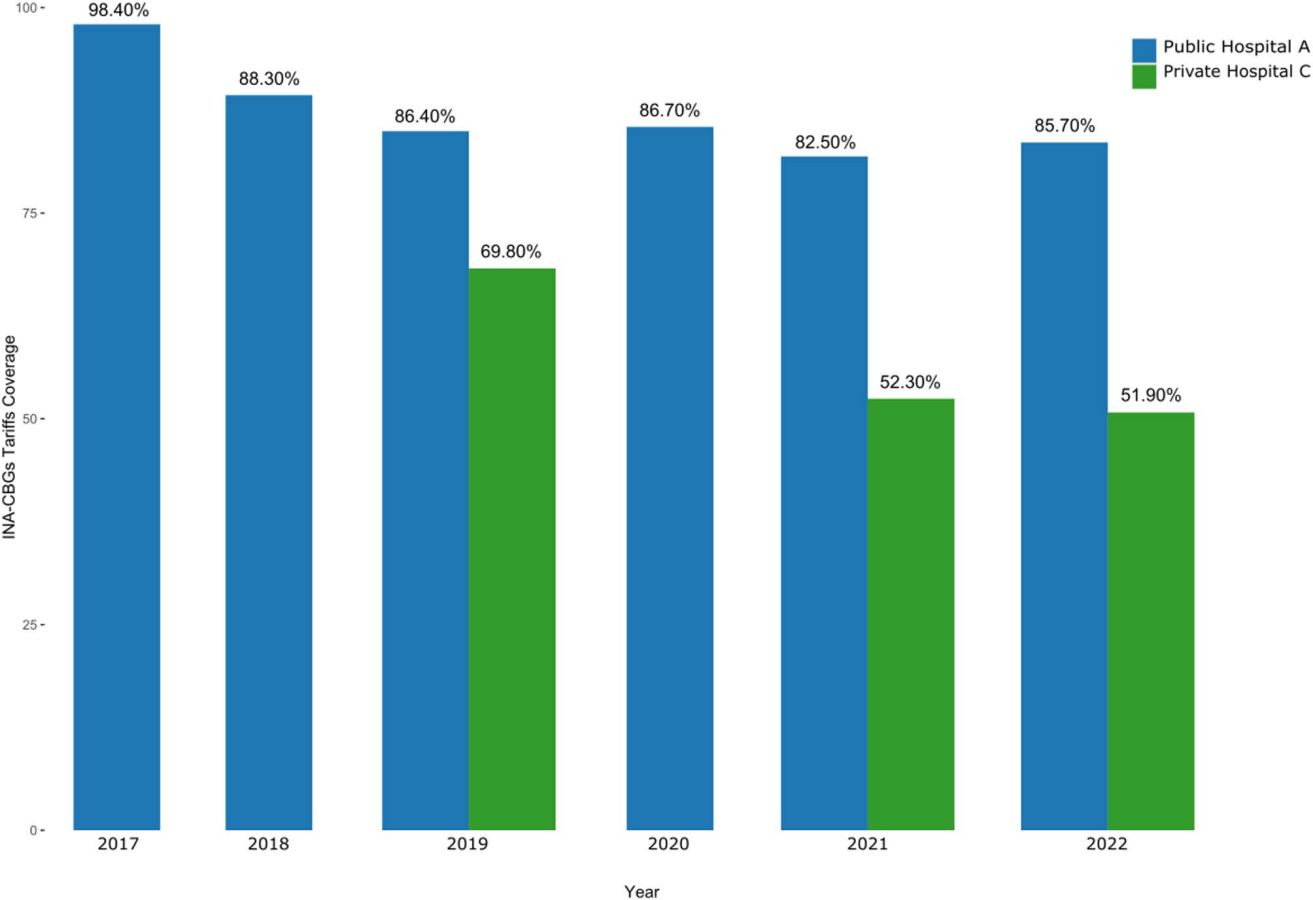
The cost-tariffs ratio of INA-CBGs tariffs of the actual costs of breast cancer outpatients with radiotherapy in both hospitals

The cost-tariffs ratio decreased gradually each year in Public Hospital A and Private Hospital C (Fig. 4), resulting in the insufficient INA-CBGs tariffs on coverage of the actual costs, with 86.85% coverage of total actual costs in Public Hospital A, and 59.07% in Private Hospital C through the review period.

### Cost components

**Fig. 5:**
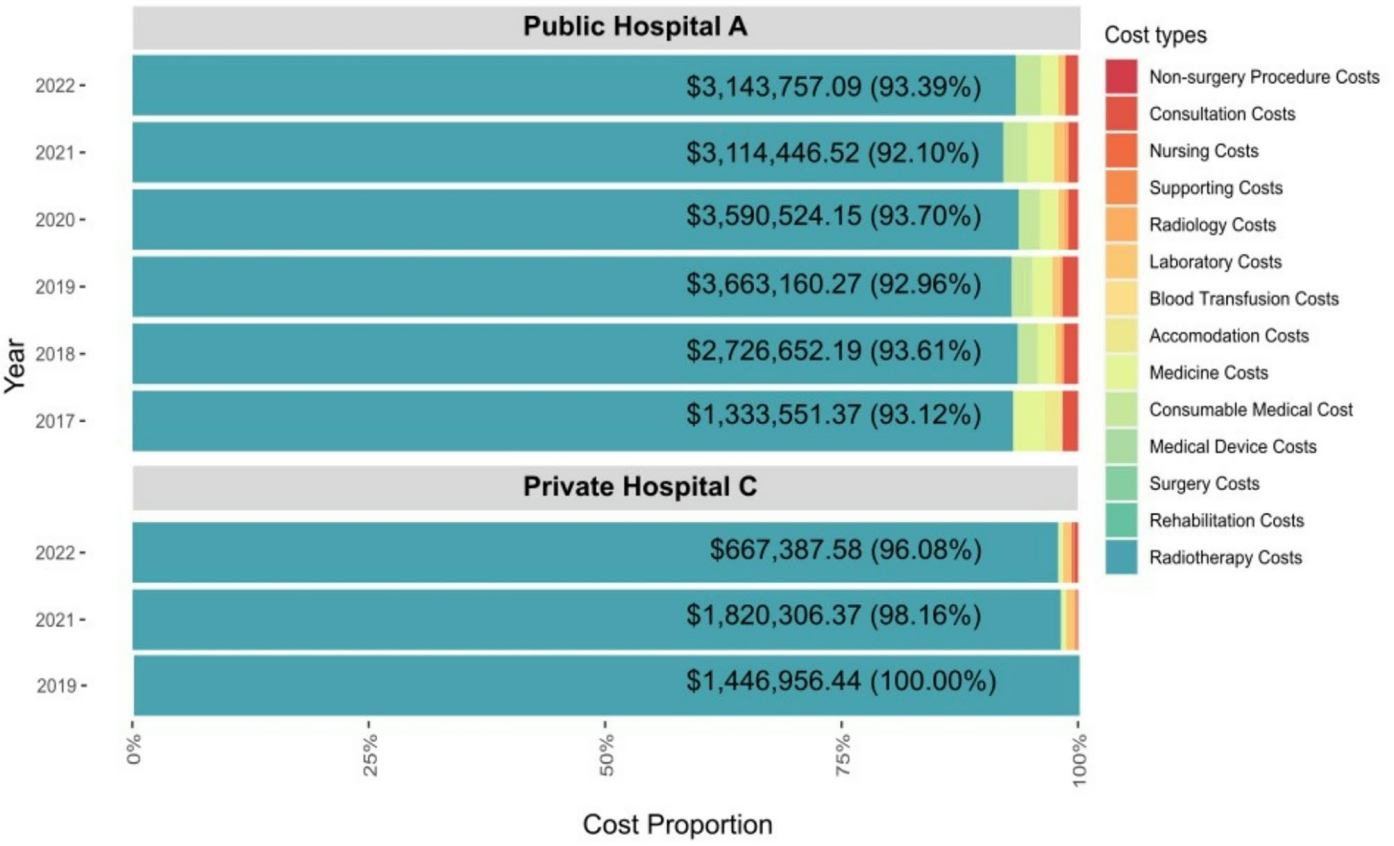
Cost components of actual costs for radiotherapy in breast cancer outpatients at Public Hospital A and Private Hospital C from 2017–2022

Radiotherapy procedures alone accounted for the majority of total actual costs under the C-3-10-0 claims data code across both hospitals, with total costs of USD 17,572,091.59 (92.1–93.7%) in Public Hospital A and USD 3,934,650.39 (97.9–100%) in Private Hospital C from 2017 to 2022. The second rank of cost component in Public Hospital A was the cost of medical consumables (2.2%), while in Private Hospital C, the laboratory costs (0.5%). Further details on the actual cost components are presented in Fig. 5.

### Sensitivity analysis

Based on our sensitivity analysis, even with the adjustment of INA-CBGs tariffs annually by the inflation rate, the tariffs remained insufficient to cover the actual costs. The tariffs could cover the actual costs after an adjustment using the inflation rate and a 25% increase from 2016’s tariffs per visit per year in Public Hospital A. However, in Private Hospital C, the INA-CBGs tariffs should be increased to 95% per visit per year (Supplementary Materials: Table 3). The discrepancies between the INA-CBGs tariffs and actual costs in age and insurance groups were significantly different, especially in Public Hospital A (*p* < 0.05; Supplementary Materials: Tables 4 and 5).

## Discussion

This study found that INA-CBGs tariffs were insufficient to cover the actual costs of radiotherapy for breast cancer in both Public and Private Hospitals. INA-CBGs tariffs only covered about 87% of all actual costs in Public and 59% in Private Hospitals, causing a consistent financial loss. BPJS Kesehatan has not increased INA-CBGs tariffs for radiotherapy procedures in breast cancer in their latest decree, and if this remains unchanged, it will substantially increase the hospital’s economic burden. Furthermore, our sensitivity analysis revealed that to fully cover the costs of radiotherapy services, the INA-CBGs tariffs need to be increased using the inflation rate and an additional increase of 25% in Public Hospital A and 95% in Private Hospital C annually. The adjustment of the INA-CBGs tariffs should be evaluated annually. Additionally, in our sensitivity analyses, the actual costs and cost differences between age groups and insurance groups have been found to be significant. It means that the age and insurance groups have contributed to the actual costs of radiotherapy services. It is crucial for hospitals to receive adequate reimbursement to not only maintain and upgrade their radiotherapy machines with new technologies but also invest in human resources to deliver high-quality radiotherapy services. The discrepancy between the actual costs and the tariffs will likely negatively impact the sustainability, accessibility, and availability of radiotherapy services [16], which could result in suboptimal treatment outcomes for breast cancer patients in the future.

Based on our findings, the most concerning results were observed in Private Hospital C, wherein the cost difference per patient and visit was higher than in Public Hospital A. This might be explained by the differences in INA-CBGs tariffs between Public Hospital A and Private Hospital C, even though they used similar radiotherapy machine resources to deliver services [10, 12, 17–19]. INA-CBGs tariffs are determined based on the mean base rate, which is calculated based on the average hospital expenses [20]. Our findings also showed that most of the actual costs were allocated for radiotherapy procedures alone, accounting for more than 90% of the total actual costs.

The higher cost difference in Private Hospital C might be due to the difference in operating costs and level of services in Private Hospital C compared to Public Hospital A [21]. Additionally, it is important to note that the data from Private Hospital C were incomplete; there were missing data from 2017, 2018, and 2020. The missing data in those years is likely caused by a non-operable machine, migration of manual documentation to electronics, or a temporary pause in cooperation between Private Hospital C and BPJS Kesehatan. As a result, the patients were referred to the public hospitals during those years. Another study about missing data in the national cancer database in the United States showed that missing data might also be caused by not well documenting the information needed for treatment while gathering the information from the patients through interviews, or the patient’s care was fragmented between hospitals [22]. However, it is unlikely this was the reason for the missing data in our study, as all data from Private Hospital C on costs were submitted directly by the hospital to the researcher, and they confirmed their dataset’s validity.

Our findings align with other Indonesian studies that observed discrepancies between INA-CBGs tariffs and actual costs of non-chemotherapy (such as laboratory, medical consumables, consultation, etc.) and chemotherapy treatments for various cancers [11, 21]. The study found that there was a negative discrepancy between the actual costs and INA-CBGs tariffs in C-4-13 I, C-4-13-II, and C-4-13-III [11]. Nonetheless, insufficient reimbursement of healthcare services has not only been reported in low-middle development index scores countries but also in high-development index scores countries. For example, in the United States of America, which reported that Medicare only covered 84% of actual costs [23].

Concerns regarding the reimbursement of radiotherapy have also been reported across European countries. These nations apply various radiotherapy reimbursement systems, such as fee-for-service, as well as budgeted and bundled payments [8]. The type of reimbursement system seems to have an impact on how radiotherapy is delivered to patients [24]. A possible solution to reduce costs is hypofractionation radiotherapy, a form of radiotherapy in which the total radiation dose is divided into fewer fractions of a larger dose, allowing for a shorter treatment period than standard radiotherapy. However, reimbursement of radiotherapy is generally based on the number of fractions delivered. Compared to conventional radiotherapy, hypofractionation has been found to be related to a financial loss for healthcare providers of about 5–10% to 30–40% but allows for reduced economic burden from the payer’s perspective [25]. Indonesia has implemented a prospective payment system in which radiotherapy tariffs are set on a per-patient, per-fraction basis. Consequently, the adoption of hypofractionation in Indonesia will increase the financial loss of the hospitals due to a lower number of fractions than conventional radiotherapy.

Several countries have implemented a copayment system for radiotherapy services and additional charges for hypofractionation, such as Korea and Japan [26]. However, this is considered too challenging to implement in Indonesia as an alternative reimbursement system, because of the financial burden experienced by the patients that comes with this system [27]. Alternative reimbursement systems, such as episode-bound payments, may provide more value for all stakeholders. It is an approximation of bundled payment that has limitations on the time and treatment. It has been introduced in the United States of America for radiotherapy reimbursement, encompassing all activities related to radiotherapy, starting from the initial consultation until immediate follow-up consultation for examining the short-term outcomes [28]. A 90-day period has been applied to finance episodes of care of radiotherapy in Medicare with short-term outcomes such as radiotherapy toxicity or patient satisfaction [29]. If the tariffs exceed the actual costs of radiotherapy services, the hospitals/healthcare providers keep the surplus; however, if the actual costs exceed the tariffs, they will not receive extra reimbursement, and the patients will not be charged for the difference [29, 30].

Moreover, in radiotherapy, reimbursement should also account for activities outside the radiotherapy sessions, starting from the initial consultation, planning, and delivery of therapy, to the management of immediate follow-up consultations to measure short-term outcomes [8]. In Indonesia, radiotherapy reimbursement is solely based on patient visits in which the patient receives radiotherapy, thereby excluding the indirect activities that contribute to the delivery of radiotherapy services, such as planning for radiotherapy or machine maintenance.

We observed a fluctuation in visits to radiotherapy centers for breast cancer treatment, which may have various underlying causes, including the COVID-19 pandemic and machine maintenance issues. Restrictions imposed by the Indonesian government during the COVID-19 pandemic reduced people’s mobility and ability to leave home, including going to the hospital [31, 32]. Moreover, patients may have been hesitant to go to the hospital due to the fear of contracting COVID-19.

### Limitations and strengths

To our knowledge, this is the first study using real-world reimbursement data to evaluate the discrepancy between INA-CBGs tariffs and actual costs of radiotherapy for outpatient breast cancer patients in Indonesia. Even though there are missing data in Private Hospital C that was not clearly acknowledged, because we only received the available data in the years 2019, 2021, and 2022. Moreover, our study only included two hospitals, which might not represent all of the hospitals in Indonesia. We were unable to collect the indirect costs (e.g., productivity losses) since we only collected the claims data from the hospitals (i.e., direct health care costs). However, the claims data from Public Hospital A and Private Hospital C enriched the description of actual costs across different hospital types and classes. Future research should elaborate on indirect costs to describe the real conditions of cost reimbursement and indirect costs, which allow for an accurate assessment of the actual costs of a particular disease or condition and can inform policy decisions to improve access and affordability of healthcare services.

Ideally, we would have included a substantial number of hospitals to represent the whole of Indonesia, but because of time and resource constrain, we limited our study to two hospitals. However, the choice of hospitals was based on the provinces with the highest prevalence of breast cancer in Indonesia: age-standardized incidence rates of breast cancer in Indonesia at 41.8 per 100,000 inhabitants and 41.4 per 100,000 in Yogyakarta Special Province [3, 33, 34]. Moreover, we chose two hospitals of a different type (one public type A and one private type C) to represent a broader spectrum of Indonesian hospitals. It would be valuable for researchers, policymakers, and other stakeholders if Indonesia could have a comprehensive cancer management/registry that linked all hospitals. This would make the patient’s journey, and all the data needed for further intervention by the policymakers could be easier to retrieve. Moreover, it would make it possible to explore differences between hospital classes and regions.

## Conclusion

For both hospitals, the INA-CBGs tariffs for outpatient radiotherapy services for breast cancer were insufficient to fully cover the actual costs during the review period. Furthermore, the difference between the tariffs and the actual costs increased over the years, emphasizing the need for revision of the C-3-10-0 tariffs. This is crucial to ensure coverage of all the actual costs in Indonesian hospitals, and to assure the sustainability, accessibility, and availability of radiotherapy treatment for breast cancer patients.

## Supplementary Information

Supplementary Material 1.

## Data Availability

The data that support the findings of this study are not openly available due to reasons of sensitivity and are available from the corresponding author upon reasonable request. Data are, however, available from the authors upon reasonable request and with permission from the Sardjito Hospital and Ken Saras Hospital.

## Abbreviations

INA-CBGs: Indonesian Case-Based Groups
NHIP: National Health Insurance Program
BPJS Kesehatan: Badan Penyelenggara Jaminan Sosial Kesehatan
ICD-9: International Classification of Disease-9th Revision
ICD-10: International Classification of Disease-10th Revision
STROBE: Strengthening the Reporting of Observational Studies in Epidemiology
C50.9: Malignant neoplasm of breast unspecified (ICD-10)
Z51.0: Radiotherapy sessions (ICD-10)
C-3-10-0: Radiotherapy procedures for outpatients
IDR: Indonesian Rupiah
USD: United States Dollar
PPP: Purchasing Power Parity
C-4-13 I, C-4-13-II, and C-4-13-III: Inpatient chemotherapy procedures with level of severity I (mild), II (moderate), and III (severe)
COVID-19: Coronavirus disease
