# Supplementary material for: Costs analysis of radiotherapy for breast cancer in Indonesia: a comparison between reimbursement tariffs and actual costs
Source: BMC Health Serv Res. 2025 May 28;25:766. doi: 10.1186/s12913-025-12849-9 (PMC12117771; doi:10.1186/s12913-025-12849-9)
Supplement: Supplementary file 1 — Supplementary Material 1. [file 12913_2025_12849_MOESM1_ESM.docx]

**Supplementary Materials**

**Table 1**. The total actual cost versus INA-CBGs tariffs for Public Hospital A and Private Hospital C from 2017-2022

| **Year** | **Public Hospital A** | | | | | **Private Hospital C** | | | | |
| --- | --- | --- | --- | --- | --- | --- | --- | --- | --- | --- |
|  | **N** | **Total INA-CBGs tariffs (USD)** | **Total actual cost (USD)** | **Cost difference (USD)** | **p-value** | **N** | **Total INA-CBGs tariffs (USD)** | **Total actual cost (USD)** | **Cost difference (USD)** | **p-value** |
| **Total** | **69,774** | **16,525,995.00** | **19,028,791.17** | **-2,502,796.17** | **<.05*** | **19,670** | **3,118,848.40** | **5,279,980.74** | **-2,161,132.34** | **<.05*** |
| 2017 | 5,895 | 1,418,418.20 | 1,441,435.25 | -23,017.05 | <.05* | NA | NA | NA | NA | NA |
| 2018 | 11,265 | 2,667,094.10 | 3,020,593.44 | -353,499.34 | <.05* | NA | NA | NA | NA | NA |
| 2019 | 14,467 | 3,415,411.10 | 3,951,931.08 | -536,519.98 | <.05* | 9,121 | 1,446,956.40 | 2,073,941.54 | -626,985.14 | <.05* |
| 2020 | 13,938 | 3,329,287.00 | 3,841,484.76 | -512,197.76 | <.05* | NA | NA | NA | NA | NA |
| 2021 | 11,756 | 2,797,912.50 | 3,391,400.50 | -593,488.00 | <.05* | 6,281 | 1,004,504.40 | 1,920,994.03 | -916,489.63 | <.05* |
| 2022 | 12,453 | 2,897,872.10 | 3,381,946,14 | -484,074.04 | <.05* | 4,268 | 667,387.60 | 1,285,045,17 | -617,657.57 | <.05* |

Abbreviations: INA-CBGs: Indonesia Case-Based Groups; USD: US dollars; N: Number of visits; NA: not available; *significant

**Table 2.** Comparison of average and median of actual cost versus the INA-CBGs tariffs per visit and per patient for Public Hospital A and Private Hospital C

|  | **INA-CBGs tariffs^a^** | | **Actual costs^a^** | | **Cost difference^a^** | |
| --- | --- | --- | --- | --- | --- | --- |
|  | **Mean ± SD** | **Median [IQR]** | **Mean ± SD** | **Median IQR** | **Mean ± SD** | **Median IQR** |
| Cost per patient |  |  |  |  |  |  |
| Public Hospital A | 4,960.00 (2,330.00) | 5,820.00 (3,093.98;6,846.40) | 5,710.00 (2,750.00) | 6,560.00 [3,679.81;7,518.46] | -752.00 (837.00) | -793.00 (-1,162.04;-98.61) |
| Private Hospital C | 2,910.00 (2,210.00) | 2,970.00 [475.92;4,847.47] | 4,920.00 (3,820.00) | 5,110.00 [839.15;7,552.34] | -2,010.00 (1,760.00) | -1,730.00 [-3,210.63;-370.19] |
| Cost per visit |  |  |  |  |  |  |
| Public Hospital A | 237.00 (2.32) | 237.00 [236.08;238.86] | 273.00 (99.10) | 272.00 [253.16;274.47] | -35.9 (99.20) | -35.30 [-35.60;-20.46] |
| Private Hospital C | 159.00 (1.28) | 159.00 [158.64;159.93] | 268.00 (72.70) | 272.00 [211.31;305.50] | -110.00 (72.70) | -112.00 [-145.57;-52.70] |

^a^ in GDP adjusted, US dollars. Abbreviations: INA-CBGs: Indonesia Case-Based Groups; SD: standard deviation; IQR: interquartile. All the tariffs and costs were in USD (US dollars).

**Table 3.** Sensitivity analysis with an increase INA-CBGs tariffs^a,b^, and inflation rate

|  | **Public Hospital A** | | | | | **Private Hospital C** | | | | | |
| --- | --- | --- | --- | --- | --- | --- | --- | --- | --- | --- | --- |
|  | **Per patient** | | **Per visit** | | **Per patient** | | | **Per visit** | | |  |
|  | **Mean (SD)** | **Median [IQR]** | **Mean (SD)** | **Median [IQR]** | **Mean (SD)** | | **Median [IQR]** | **Mean (SD)** | **Median [IQR]** |  |  |
| **2017** | | | | | | | | | | |  |
| INA-CBGs tariffs | 5,580.00 (3,280.00) | 6,770.00 [2,706.91; 8,421.48] | 301.00 (0) | 301.00 [300.77;300.77] | NA | | NA | NA | NA |  |  |
| Actual costs | 4,530.00 (2,750.00) | 5,050 .00 [2,283.09;6,792.2] | 245.00 (141.00) | 228.00 [227.70;300.77] | NA | | NA | NA | NA |  |  |
| Cost difference | 1,040 .00 (1,220.00) | 714 .00 [243.99;1,739.76] | 56.20 (141.00) | 73.10 [24.28;73.06] | NA | | NA | NA | NA |  |  |
| **2018** | | | | | | | | | | |  |
| INA-CBGs tariffs | 6,090.00 (2,940.00) | 7,100.00 [3,551.39; 8,582.53] | 296.00 (0) | 296.00 [295.95:295.95] | NA | | NA | NA | NA |  |  |
| Actual costs | 5,520.00 (2,710.00) | 6,450.00 [3,218.54; 7,338.93] | 268.00 (85.60) | 272.00 [233.78;272.05] | NA | | NA | NA | NA |  |  |
| Cost difference | 573.00 (772.00) | 430.00 [149.05;865.25] | 27.80 (85.60) | 23.90 [23.90;62.17] | NA | | NA | NA | NA |  |  |
| **2019** | | | | | | | | | | |  |
| INA-CBGs tariffs | 5,870.00 (2,860.00) | 7,080.00 [3,541.24; 7,672.69] | 295.00 (0) | 295.00 [295.10;295.10] | 5,080.00 (4,340.00) | | 4,950.00 [309.35; 9,280.45] | 309.00 (0) | 309.00 [309.35;309.35] |  |  |
| Actual costs | 5,440.00 (2,670.00) | 6,110.00 [3,577.12; 7,004.89] | 273.00 (95.80) | 271.00 [247.14;280.97] | 3,740.00 (3,180.00) | | 3,530.00 [299.15; 6,604.15] | 227.00 (72.40) | 203.00 [185.20;254.19] |  |  |
| Cost difference | 436.00 (753.00) | 357.00 [118.63;532.26] | 21.90 (95.80) | 23.80 [14.13;47.96] | 1,350.00 (1,240.00) | | 1,120.00 [123.11; 2,627.29] | 82.00 (72.40) | 106.00 [55.16;124.15] |  |  |
| **2020** | | | | | | | | | | |  |
| INA-CBGs tariffs | 6,460.00 (2,910.00) | 7,460.00 [4,702.64; 8,658.82] | 299.00 (0) | 299.0 [298.58;298.58] | NA | | NA | NA | NA |  |  |
| Actual costs | 5,970.00 (2,730.00) | 6,890.00 [4,656.69; 7,482.36] | 276.00 (88.70) | 274.00 [274.47;274.47] | NA | | NA | NA | NA |  |  |
| Cost difference | 497.00 (775.00) | 460.00 [144.77;630.86] | 23.00 (88.70) | 24.10 [24.11;24.11] | NA | | NA | NA | NA |  |  |
| **2021** | | | | | | | | | | |  |
| INA-CBGs tariffs | 6,600.00 (2,890.00) | 7,440.00 [5,057.47; 8,627.45] | 297.00 (0) | 297.00 [297.50;297.50] | 6,600.00 (4,370.00) | | 6,550.00 [2,494.87; 9,979.47] | 312.00 (0) | 312.00 [311.86;311.86] |  |  |
| Actual costs | 6,400.00 (2,920.00) | 6,990.00 [4,838.74;8,265.60] | 288.00 (111.00) | 273.00 [273.48;273.48] | 6,470.00 (4,200.00) | | 6,550.00 [2,799.04; 9,572.02] | 306.00 (49.50) | 295.00 [271.55;339.44] |  |  |
| Cost difference | 200.00 (711.00) | 370.00 [44.60;512.64] | 9.02 (111.00) | 24.00 [24.02;24.02] | 127.00 (439.00) | | 57.30 [-164.17; 472.50] | 6.02 (49.50) | 17.30 [-27.58;40.31] |  |  |
|  |  |  |  |  |  | |  |  |  |  |  |
|  |  |  |  |  |  | |  |  |  |  |  |
| **Table 3.** Sensitivity analysis with an increase INA-CBGs tariffs^a,b^, and inflation rate (continued) | | | | | | | | | |  |  |
| **2022** | | | | | | | | | | |  |
| INA-CBGs tariffs | 6,420.00 (2,650.00) | 7,270.00 [4,944.98;8,508.27] | 291.00 (0) | 291.00 [290.88;290.88] | 5,890.00 (3,950.00) | | 6,100.00 [2,134.45; 9,452.57] | 305.00 (0) | 305.00 [304.92;304.92] |  |  |
| Actual costs | 6,000.00 (2,500.00) | 6,510.00 [4,630.04;7,737.71] | 272.00 (85.50) | 253.00 [253.16;267.39] | 5,810.00 (3,800.00) | | 6,110.00 [2,377.25; 8,985.42] | 301.00 (53.10) | 292.00 [265.51;331.89] |  |  |
| Cost difference | 426.00 (666.00) | 555.00 [226.62;800.58] | 19.30 (85.50) | 37.70 [23.49;37.72] | 74.00 (379.00) | | 31.00 [-195.23; 435.77] | 3.83 (53.10) | 12.60 [-26.97;39.41] |  |  |
| **Overall** | | | | | | | | | |  |  |
| INA-CBGs tariffs | 6,200.00 (2,910.00) | 7,270.00 [3,867.48; 8,558.00] | 296.00 (2.91) | 296.00 [295.10;298.58] | 5,670.00 (4,320.00) | | 5,790.00 [928.05; 9,452.57] | 309.00 (2.50) | 309.00 [309.35;311.86] |  |  |
| Actual costs | 5,710.00 (2,750.00) | 6,560.00 [3,679.81;7,518.46] | 273.00 (99.10) | 272.00 [253.16;274.47] | 4,920.00 (3,820.00) | | 5,110.00 [839.15; 7,552.34] | 268.00 (72.70) | 272.00 [211.31;305.50] |  |  |
| Cost difference | 489.00 (824.00) | 429.00 [143.23;726.91] | 23.30 (99.20) | 24.10 [23.81;38.58] | 747.00 (1,120.00) | | 269.00 [14.62; 1,307.21] | 40.80 (72.70) | 39.40 [6.22;98.01] |  |  |

a: increase INA-CBGs tariffs by 25% in Public Hospital A. b: increase INA-CBGs tariffs by 95% in Private Hospital C. Abbreviation: NA: Not available. All the tariffs and costs were in USD (US dollars).

**Table 4.** Sensitivity analysis costs by age groups (<65 years and ≥65 years)

| **<65 years** | | | | | | | **≥65 years** | | | | | | |
| --- | --- | --- | --- | --- | --- | --- | --- | --- | --- | --- | --- | --- | --- |
|  | **INA-CBGs tariffs** | | **Actual costs** | | **Cost difference** | | **INA-CBGs tariffs** | | **Actual costs** | | | **Cost difference** | |
|  | **Mean (SD)** | **Median (IQR)** | **Mean (SD)** | **Median (IQR)** | **Mean (SD)** | **Median (IQR)** | **Mean (SD)** | **Median (IQR)** | **Mean (SD)** | **Median (IQR)** | **Mean (SD)** | | **Median (IQR)** |
| **2017** | | | | | | | | | | | | | |
| Public Hospital A | 241.00 (0) | 241.00 [240.62; 240.62] | 243.00 (127.00) | 228.00 [227.71; 276.48] | -2.89 (127.00) | 12.90 [-35.87; 12.91]^a^ | 241.00 (0) | 241.00 [240.62; 240.62] | 254.00 (232.00) | 228.00 [227.70; 261.77] | -13.40 (232.00) | | 12.90 [-21.15; 12.91]^a^ |
| Private Hospital C | NA | NA | NA | NA | NA | NA | NA | NA | NA | NA | NA | | NA |
| **2018** | | | | | | | | | | | | | |
| Public Hospital A | 237.00 (0) | 237.00 [236.76; 236.76] | 268.00 (88.30) | 272.00 [233.78; 272.05] | -31.60 (88.30) | -35.30 [-35.30; 2.98] | 237.00 (0) | 237.00 [236.76; 236.76] | 266.00 (51.70) | 272.00 [224.06; 272.05] | -28.80 (51.70) | | -35.30 [-35.30; 12.71] |
| Private Hospital C | NA | NA | NA | NA | NA | NA | NA | NA | NA | NA | NA | | NA |
| **2019** | | | | | | | | | | | | | |
| Public Hospital A | 236.00 (0) | 236.00 [236.09; 236.09] | 274.00 (98.50) | 271.00 [247.14; 280.97] | -37.70 (98.50) | -35.20 [-44.89; -11.06]^b^ | 236.00 (0) | 236.00 [236.09; 236.09] | 267.00 (65.40) | 271.00 [223.42; 280.98] | -31.30 (65.40) | | -35.20 [-44.89; 12.67]^b^ |
| Private Hospital C | 159.00 (0) | 159.00 [158.64; 158.64] | 228.00 (71.30) | 203.00 [186.79; 254.26] | -68.90 (71.30) | -44.90 [-95.63; -28.15] | 159.00 (0) | 159.00 [158.64; 158.64] | 225.00 (85.50) | 199.00 [178.73; 252.53] | -66.60 (85.50) | | -40.70 [-93.89; -20.08] |
| **2020** | | | | | | | | | | | | | |
| Public Hospital A | 239.00 (0) | 239.00 [238.86; 238.86] | 275.00 (88.20) | 274.00 [274.47; 274.47] | -36.40 (88.20) | -35.60 [-35.61; -35.61]^c^ | 239.00 (0) | 239.00 [238.86; 238.86] | 279.00 (93.00) | 274.00 [274.47; 274.47] | -40.30 (93.00) | | -35.60 [-35.61; -35.61]^c^ |
| Private Hospital C | NA | NA | NA | NA | NA | NA | NA | NA | NA | NA | NA | | NA |
| **2021** | | | | | | | | | | | | | |
| Public Hospital A | 238.00 (0) | 238.00 [238.00; 238.00] | 288.00 (105.00) | 273.00 [273.48; 273.48] | -49.80 (105.00) | -35.50 [-35.48; -35.48]^d^ | 238.00 (0) | 238.00 [238.00; 238.00] | 295.00 (155.00) | 273.00 [273.48; 282.84] | -56.60 (155.00) | | -35.50 [-44.84; -35.48]^d^ |
| Private Hospital C | 160.00 (0) | 160.00 [159.93; 159.93] | 306.00 (49.60) | 294.00 [271.55; 339.44] | -146.00 (49.60) | -134.00 [-179.51; -111.63] | 160.00 (0) | 160.00 [159.93; 159.93] | 304.00 (48.10) | 299.00 [271.55; 326.31] | -145.00 (48.10) | | -139.00 [-166.39; -111.63] |
| **2022** | | | | | | | | | | | | | |
| Public Hospital A | 233.00 (0) | 233.00 [232.71; 232.71] | 271.00 (79.70) | 253.00 [253.16; 267.39] | -38.40 (79.70) | -20.50 [-34.69; -20.46]^e^ | 233.00 (0) | 233.00 [232.71; 232.71] | 275.00 (114.00) | 253.00 [253.16; 268.31] | -41.80 (114.00) | | -20.50 [-35.61; -20.46]^e^ |
| Private Hospital C | 156.00 (0) | 156.00 [156.37; 156.37] | 301.00 (53.40) | 289.00 [265.51; 331.89] | -144.00 (53.40) | -133.00 [-175.52; -109.14]^f^ | 156.00 (0) | 156.00 [156.37; 156.37] | 303.00 (50.40) | 299.00 [265.51; 331.89] | -147.00 (50.40) | | -142.00 [-175.52; -109.14]^f^ |
| **Overall** | | | | | | | | | | | | | |
| Public Hospital A | 237.00 (2.30) | 237.00 [236.08; 238.86] | 273.00 (96.50) | 272.00 [253.16; 274.47] | -35.60 (96.60) | -35.30 [-35.61-20.46] | 237.00 (2.50) | 237.00 [236.08; 238.86] | 274.00 (119.00) | 271.00 [253.16; 274.47] | -37.80 (119.00) | | -35.20 [-35.61; -20.46] |
| Private Hospital C | 159.00 (1.26) | 159.00 [158.64; 159.93] | 268.00 (72.40) | 272.00 [210.10; 305.50] | -109.00 (72.40) | -112.00 [-145.57; -51.57] | 159.00 (1.26) | 159.00 [158.64; 159.93] | 273.00 (76.30) | 272.00 [221.52; 305.50] | -115.00 (76.50) | | -112.00 [-146.57; -62.88] |

^a,b,c,d,e,f^: comparison of cost difference between age <65 years and ≥65 with p<0.05. Abbreviation: SD: standard deviation; NA: Not available. All the tariffs and costs were in USD (US dollars).

**Table 5.** Sensitivity analysis costs by insurance class groups (class 2 and class 3) in Public Hospital A

|  |  | **2017** | **2018** | **2019** | **2020** | **2021** | **2022** | **Overall** | | |
| --- | --- | --- | --- | --- | --- | --- | --- | --- | --- | --- |
|  |  | **Class 2** | **Class 3** | **Class 3** | **Class 3** | **Class 3** | **Class 3** | **Class 2** | **Class 3** | **p-value** |
| INA-CBGs tariffs | Mean (SD) | 241.00 (0) | 237.00 (0) | 236.00 (0) | 239.00 (0) | 238.00 (0) | 233.00 (0) | 241.00 (0) | 237.00 (2.12) |  |
|  | Median [IQR] | 241.00 [240.62; 240.62] | 237.00 [236.76; 236.76] | 236.00 [236.08; 236.08] | 239.00 [238.86; 238.86] | 238.00 [238.00; 238.00] | 233.00 [232.70; 232.70] | 241.00 [240.61; 240.61] | 237.00 [236.0829; 237.9987] |  |
| Actual costs | Mean (SD) | 245.00 (141.00) | 268.00 (85.60) | 273.00 (95.80) | 276.00 (88.70) | 288.00 (111.00) | 272.00 (85.50) | 245.00 (141.00) | 275.00 (93.90) | <.05* |
|  | Median [IQR] | 228.00 [227.70; 276.48] | 272.00 [233.78; 272.05] | 271.00 [247.14; 280.97] | 274.00 [274.47; 274.47] | 273.00 [273.48; 273.48] | 253.00 [253.16; 267.39] | 228.00 [227.70; 276.48] | 272.00 [253.16; 274.47] |  |
| Cost difference | Mean (SD) | -3.90 (141.00) | -31.40 (85.60) | -37.10 (95.80) | -36.70 (88.70) | -50.50 (111.00) | -38.90 (85.50) | -3.90 (141.00) | -38.80 (93.90) | <.05* |
|  | Median [IQR] | 12.90 [-35.87; 12.91] | -35.30 [-35.29; 2.98] | -35.20 [-44.89; -11.06] | -35.60 [-35.61; -35.61] | -35.50 [-35.48; -35.48] | -20.50 [-34.69; -20.46] | 12.90 [-35.87; 12.91] | -35.30 [-35.61; -20.46] |  |

Abbreviation: SD: standard deviation; NA: Not available; USD: US dollars; *significant
